# Predicting nurse burnout: A logistic regression approach to workplace and demographic factors

**DOI:** 10.1016/j.dialog.2025.100267

**Published:** 2025-12-21

**Authors:** Edona Haxhija, Drita Kruja, Zamira Shabani

**Affiliations:** a European University of Tirana, Tirana, Albania; b Department of Nursing, Faculty of Nursing, “AAB College”, 10000, Prishtina, Republic of Kosovo; c Center for Sustainable Development, Faculty of Economy, Business and Development, European University of Tirana, Tirana, Albania; d Department of Clinical Subjects, Faculty of Natural Sciences, University of Shkodra “Luigj Gurakuqi”,” 4001 Shkodër, Albania

**Keywords:** Healthcare workforce, Job satisfaction, Nurse burnout, Professional development, Supervisor support, Rural nurses, Workload

## Abstract

**Purpose:**

This study aimed to identify key occupational and demographic factors associated with nurse burnout in a major public hospital in Albania.

**Methods:**

A descriptive cross-sectional survey was conducted among nurses in a regional hospital. Nursing management invited all units to participate. Nurses completed the questionnaire voluntarily and anonymously during breaks. The survey included job satisfaction, burnout risk, working conditions, supervisor and colleague support, workload, shift duration, career opportunities, and demographic variables. Cluster analysis was used to categorize nurses, and exploratory factor analysis examined the structure of job satisfaction factors.

**Results:**

Data from 345 nurses showed that high workload and long shifts significantly increased burnout risk. Strong supervisor support and greater job satisfaction were associated with reduced burnout. Nurses in rural settings had 1.57 times higher odds of burnout compared to urban nurses. Female nurses had 1.25 times greater odds of burnout than male nurses. Advanced education and better career advancement opportunities were linked to lower burnout levels.

**Conclusion:**

Burnout is more prevalent among rural nurses and, to a lesser extent, among female nurses, suggesting the need for context-sensitive and inclusive interventions. Burnout stems from systemic challenges such as excessive workload, insufficient managerial support, and role misalignment. Addressing these issues requires organizational changes including staffing improvements, supportive leadership, and professional development. Future research should apply standardized burnout measures and longitudinal approaches to better understand nurse well-being.

## Highlights

• High workload and long shifts significantly increase nurse burnout risk.
• Job satisfaction and supervisor support strongly reduce burnout levels.
• Nurses in rural hospitals show 57 % higher burnout odds than urban nurses.
• Higher education levels are linked to lower burnout and greater resilience.
• Logistic regression classified 72 % of nurses accurately by burnout level.

## 1 Introduction

Nurse burnout is a significant issue in healthcare, characterized by emotional exhaustion, depersonalization, and a reduced sense of personal success [1]. Long shifts, heavy workloads, and poor support lead to it; from this follows lower job satisfaction, absenteeism, and high turnover rates [2]. Keeping effective patient care and preserving workforce sustainability depend on managing nurse burnout [3]. Rich countries' extensive research has revealed significant reasons behind nurse burnout. Working conditions include too long hours and heavy patient loads significantly affected burnout and job retention rates, according to research analyzing data from more than 50,000 licensed nurses in the United States [4].

Research conducted during the COVID-19 epidemic in the United Kingdom also revealed that demographic variables included being female, younger, or recently allocated to a different department raised burnout risk among nurses [5]. Another UK study found that nurses who felt more supported by their companies were more resilient and showed less burnout, therefore highlighting the preventive power of workplace support networks [6]. Despite these findings, little is known about the elements causing burnout in the underdeveloped healthcare systems including Albania. In Albania, the COVID-19 outbreak worsened already existing challenges for nurses, which increased both their psychological stress and workload [7].

Previous studies on Albanian nurses have largely focused on job satisfaction and career advancement instead of burnout as such. Studies reveal that job satisfaction is closely associated with factors like compensation, chances for growth, and contacts at work; its direct impact on burnout has not been well examined [[8], [9], [10]]. A new meta-analysis showing a notable increase in burnout among European nurses throughout the epidemic strengthens the need of region-specific research even more [11].Ultimately, the outputs will enable the creation of a more sustainable and resilient nurse workforce, therefore enhancing the patient care outcomes [12,13].

This study addresses the gap by examining predictors of burnout risk among nurses in an Albanian regional hospital, using statistical modeling to integrate multiple theoretical perspectives. By analyzing how workplace conditions and individual characteristics relate to burnout, we aim to generate evidence-based recommendations for interventions to reduce nurse burnout and improve job satisfaction in this context.

## 2 Literature review

### 2.1 Burnout in nursing

Burnout in nurses is closely associated with excessive workloads, insufficient supervisory assistance, and emotional distress [14]. It elevates the probability of nurses departing from their roles [15]. Recent American studies underscore the persistent problem of nurse burnout and its adverse impact on patient satisfaction and healthcare quality. A comprehensive review and meta-analysis identified a substantial association between nurse burnout and diminished quality and safety of treatment, as well as decreased patient satisfaction [3,16,17]. Contributing elements encompass excessive workload, protracted working hours, and insufficient support. The COVID-19 pandemic further aggravated these concerns, intensifying stress and burnout among nurses [18]. A recent study conducted in European hospitals indicated that heightened burnout levels had continued after the epidemic, particularly in high-stress settings [17]. Comprehending the determinants of nurse burnout is essential for formulating tailored treatments to promote nurse well-being and enhance patient care outcomes.

### 2.2 Workplace and demographics as burnout factors

#### 2.2.1 Workload & shift length

Numerous studies have found that nurse burnout is highly influenced by excessive workloads and long working hours. A 2022 study in New Jersey hospitals observed that during the COVID-19 pandemic, nurses frequently had more patients under their care than they felt was safe, resulting in heightened burnout and an increased intention to leave the profession [19]. According to a study by Scott-Marshall [20], nurses working 12-h or longer shifts reported higher levels of burnout compared to those working shorter shifts. The gap between actual staffing levels and recommended standards was strongly associated with elevated burnout in this study. Similarly, an international survey reported that nurses working more than 50 h per week had a noticeably increased risk of burnout compared to those with more manageable hours [21]. These findings underscore that chronic understaffing and overtime are major drivers of burnout.

#### 2.2.2 Job satisfaction & supervisor support

Elevated job satisfaction mitigates emotional tiredness associated with burnout. Retention of nurses is contingent upon enhancing happiness via acknowledgment, professional advancement chances, and nurturing work settings [3]. Research demonstrates that nurses who perceive robust supervisory support manage work-related stress more effectively and have reduced levels of burnout. A 2023 study indicated that insufficient supportive supervision elevated stress and intentions to resign, but adequate support fostered resilience and mitigated burnout [22].

#### 2.2.3 Education level & career growth

The correlation between higher education and defined career growth trajectories is associated with increased job satisfaction among nurses [23]. A 2023 report from the Health Resources and Services Administration indicates that an increasing number of nurses are entering the industry with bachelor's degrees, correlating greater education with improved employment opportunities [24]. Professional development enhances morale, performance, and satisfaction, thereby reducing burnout. Nurses with continuous education or advanced degrees experience more autonomy and job control, which positively impacts patient care and personal fulfillment [25,26].

#### 2.2.4 Urban vs. rural differences

The influence of work location on burnout is intricate. Certain studies indicate that rural healthcare may provide protective elements such as strong community connections and compassion satisfaction [27]. Conversely, additional studies indicate that rural nurses experience comparable or greater levels of burnout attributable to resource deficiencies and professional isolation [28]. The closure of rural facilities exacerbates staffing shortages and increases stress in the remaining hospitals [29]. Enhanced scheduling in rural regions may alleviate burnout [30]. These varied outcomes underscore the necessity for contextually tailored interventions.

#### 2.2.5 Gender differences

Gender affects burnout, with indications that female nurses may endure marginally elevated levels owing to societal and familial role expectations and increased stress reporting. Female nurses frequently juggle professional responsibilities and familial obligations, heightening their susceptibility to burnout [31]. While differences are often small, workplace measures that cater to gender-specific requirements, such as flexible scheduling or childcare assistance, can mitigate burnout among female nurses [32].

By incorporating variables such as workload, support, education, environment, and demographics, we achieve a thorough comprehension of nurse burnout that guides the study's design and hypotheses.

### 2.3 Logistic regression in burnout prediction

Logistic regression is extensively employed in healthcare analytics to discern burnout risk factors, owing to its interpretability in contrast to intricate machine learning models (like XGBoost) [33]. Recent research illustrates its efficacy in forecasting fatigue risk when demographic and occupational characteristics are incorporated [34].

## 3 Theoretical framework and hypotheses

This study is grounded in five complementary theoretical models of burnout: the Job Demands-Resources (JD-R) model, Conservation of Resources (COR) theory, Social Exchange Theory (SET), Person-Environment Fit (P-E Fit) theory, and Self-Determination Theory (SDT). These frameworks collectively inform how different individual, organizational, and psychological factors may contribute to or alleviate burnout, shaping our hypotheses and analysis.

#### 3.1.1 Job Demands-Resources (JD-R) Model

The JD-R model asserts that burnout arises from a disparity between elevated job demands (e.g., workload, emotional strain) and inadequate job resources (e.g., support systems) [35]. A meta-analysis established that sustaining a balance between demands and resources is crucial for the well-being and retention of nurses [3].

#### 3.1.2 Conservation of Resources (COR) Theory

COR theory proposes that one of the key factors that contributes to burnout is the depletion of valuable resources [36].

#### 3.1.3 Social Exchange Theory (SET)

SET posits that employees respond to organizational support with heightened engagement and less emotional weariness [37]. Recognition, equity, and a sense of worth enhance organizational commitment and resilience [38].

#### 3.1.4 Person-Environment Fit (P-E Fit) Theory

The P-E Fit theory elucidates burnout as a result of a discordance between an individual and their work environment [39]. When a nurse's values, abilities, or expectations are at odds with the organizational culture or role requirements, it results in stress and disengagement. Aligning professional roles with individual values has demonstrated a reduction in burnout [40].

#### 3.1.5 Self-Determination Theory (SDT)

Self-Determination Theory focuses on intrinsic motivation and the fulfillment of fundamental psychological needs: autonomy, competence, and relatedness [41]. In nursing, environments that foster autonomy and professional development are associated with less burnout and increased job satisfaction [17].

**Fig. 1:**
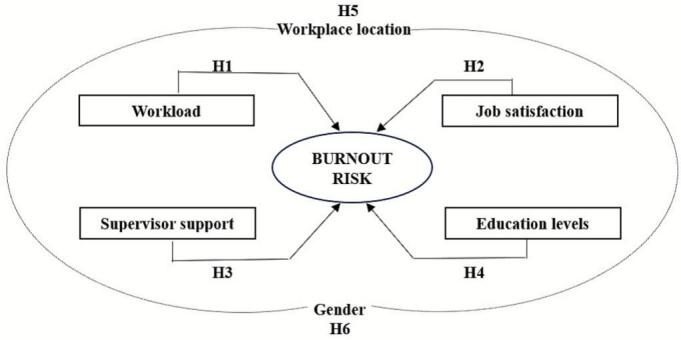
Theoretical framework of the burnout risk and organizational, individual and psychological antecedents.

Drawing on these theories, we developed a conceptual model (Fig.1) in which:

• **Organizational factors** (e.g., supervisor and colleague support, workload, shift duration, career advancement opportunities),
• **Individual factors** (e.g., age, gender, years of experience, department, location, education level),
• **Psychological factors** (e.g., feeling of appreciation and respect, overall job satisfaction) interact to influence nurse burnout risk.

Each hypothesis corresponds to one or more theoretical models, ensuring our study is grounded in existing theory while empirically testing these ideas. To address the research objectives and the theoretical framework, the subsequent hypotheses are proposed:

o **H1:** There is a significant relationship between higher workload and increased burnout among nurses. (JD-R)
o **H2:** There is a significant relationship between higher job satisfaction and lower burnout levels among nurses. (JD-R; P-E Fit; SDT)
o **H3:** There is a significant relationship between strong supervisor support and reduced burnout among nurses. (JD-R; COR; SET)

o **H4:** There is a significant relationship between higher education levels and lower burnout risk among nurses. (SDT; P-E Fit)
o **H5:** There is a significant relationship between workplace location (urban vs. rural) and burnout levels among nurses. (P-E Fit; COR)
o **H6:** There is a significant relationship between gender and burnout, with female nurses experiencing higher burnout levels than male nurses. (Empirical evidence based on literature review)

## 4 Research methodology

### 4.1 Research design

This research employed a descriptive cross-sectional survey methodology to evaluate nurse satisfaction and its related components. The survey was conducted from June to July 2024, in a prominent public hospital in Albania that serves as a regional tertiary care center. Data were gathered through a structured, self-administered online questionnaire designed to ensure anonymity. All registered nurses across various departments were eligible to participate.

The hospital environment included a wide range of units such as medical and surgical wards, critical care, emergency, pediatrics, and obstetrics offering a broad view of nursing experiences across specialties. The study setting reflects broader systemic challenges common to many healthcare systems, including limited resources, low wages, and ongoing health sector reforms that may influence job satisfaction.

### 4.2 Measures

The data for this study were collected using a 30-item Nurse Satisfaction Survey adapted from a practice-based instrument originally developed by Carepatron [42]. The survey included five key domains:

a) Workplace Relationships: Supervisor and co-worker support.
b) Workplace Conditions: Safety, facilities, tools.
c) Compensation & Development: Pay, Chances for advancement, and Work stability.
d) Workload & Leadership: Management effectiveness, Patient load, Shift duration
e) Personal Perceptions: Professional fulfillment, Pride, and Respect.

Additional background information was provided by participants regarding:

a) **Demographics**: Age, Gender, Education level, Years of experience.
b) **Job-related context**: Shift duration, Job role, and Workplace location (urban vs. rural).

Although gold-standard instruments such as the Copenhagen Burnout Inventory (CBI) [43] and Maslach Burnout Inventory (MBI) [44] offer comprehensive multidimensional assessment of burnout, their use was deemed infeasible in this context. Piloting efforts and participant feedback indicated that including full burnout scales significantly reduced engagement and increased fatigue, particularly in this high-demand hospital setting. Therefore, we adopted a pragmatic alternative approach by assessing key workplace variables such as workload, job satisfaction, and supervisor support as proxies for burnout risk. This decision is supported by prior research where indirect measures were effectively used when standard burnout inventories were impractical. Importantly, our instrument showed strong internal consistency (Cronbach's α = 0.85) and its factor structure aligned with established theoretical frameworks (JD-R, COR, P-E Fit, SDT), enhancing its construct validity. We fully acknowledge this methodological compromise as a limitation and recommend that future studies integrate a validated burnout scale for improved direct measurement and cross-study comparability.

### 4.3 Questionnaire design

The questionnaire was translated and adapted into Albanian to improve its relevance and precision for the local nursing staff. A pilot study with 20 nurses was conducted to assess clarity and effectiveness, and to measure the validity and reliability of the instrument. A panel of experts in nursing and healthcare management reviewed the items to ensure content validity and comprehensive coverage of job satisfaction dimensions.

The finalized tool consisted of 30 items divided into thematic sections, combining open-ended, closed-ended, and Likert scale questions:

1. Demographic Information (6 questions): Age, Gender, Years of experience as a nurse, Highest degree of education, Work department, Urban vs Rural location of residence.
2. Job Satisfaction (8 Likert scale questions, rated from 1 to 5): Nurses assessed their level of satisfaction with different aspects of their work environment using a five-point Likert scale ranging from 1 (Not satisfied at all) to 5 (Very satisfied). The items addressed satisfaction with various aspects of their role, including: their position as a nurse in the hospital, relationships with colleagues (junior, senior, and peers), availability of resources and equipment, leadership from the head nurse, and salary.
3. Workplace Environment Factors (16 Yes/No questions): Nurses were asked to respond Yes/No to statements related to their work environment and professional opportunities, including: workplace safety, job security, understanding and alignment with the institution's mission and vision, opportunities for advancement, adequacy of staffing, pressure due to staff shortages, difficulty dealing with patients and their relatives, support from the head nurse and colleagues, respect from peers and supervisors, whether management listens to their concerns, and pride in their work and profession.

All Likert scale items were coded such that higher scores reflect more positive evaluations (higher satisfaction, better support, etc.), and Yes/No items were coded as binary indicators of whether a potential stressor or resource was present.

The full questionnaire is included in Appendix A.

### 4.4 Sampling

This study was carried out in the Regional Hospital of Shkodra, one of Albania's six major public hospitals and the primary referral center for the northern region. The hospital was chosen because it demonstrates a typical public healthcare facility in Albania, achieving national criteria in staffing, funding, and treatment provision while also dealing with common systemic issues such as manpower shortages, severe workloads, and inadequate resources.

At the time data were collected, the hospital employed 430 nurses. Following formal consent from the hospital administration, 345 nurses volunteered to complete an anonymous online survey, which was distributed via a secure link over a two-month period. The nursing administration encouraged participation by issuing invitations to each unit, and nurses completed the survey during their work breaks. The response rate was over 80 %, meeting the sample size requirements for a 95 % confidence level and a 5 % margin of error, verifying the data's representativeness.

Nurses from approximately 19 units participated, including medical-surgical, emergency, acute care, and outpatient departments. The predominant proportion (46 %) was employed in general medical-surgical units, and the remainder was distributed among specialized wards (each accounting 1–6 % of the sample). This variability improves the generalizability of the findings (Table 1).

**Table 1.** Sample characteristics of surveyed nurses (*N* = 345).

Characteristic	Value (Mean ± SD or N (%)
Age (years)	38.1 ± 10.3 (range 20–61)
Years of experience	12.7 ± 9.9 (range 1–40)
Gender: Female	280 (81.2 %)
Gender: Male	65 (18.8 %)
Education – Nursing Diploma	186 (53.9 %)
Education – Bachelor's	126 (36.5 %)
Education – Master's	33 (9.6 %)
Works in the medical/surgical unit (largest dept)	160 (46.4 %)
Works in other specialty unit(s)	185 (53.6 %)

Table 1 summarizes the demographic characteristics of the respondents. The majority of participants were female (81 %), indicative of the standard gender distribution in nursing. Ages varied from 20 to 61 years (mean 38.1 ± 10.3), with an average work experience of 12.7 ± 9.9 years (range 1–40), reflecting a combination of early-career and experienced nurses. Educational qualifications varied: 53.9 % held a secondary-level nursing diploma, 36.5 % held a Bachelor's degree, and 9.6 % attained a Master's degree or above.

### 4.5 Data analysis

The Kaiser-Meyer-Olkin (KMO) test was used to determine the data's eligibility for factor analysis, and a value of 0.734 indicated a good degree of sampling adequacy. Bartlett's Test of Sphericity was statistically significant (χ^2^ = 1394.88, *p* < 0.001), indicating that the dataset is eligible for factor analysis.

An exploratory factor analysis (principal components, varimax rotation) identified four factors explaining 82.2 % of the total variance.

These factors were theoretically interpretable and consistent with previous models:

Factor 1 (Workplace Demands) was significantly weighted toward workload and shift-related factors, reflecting the “job demands” facet of the JD-R model.

Factor 2 (Job Satisfaction & Support) comprised measures about supervisors' and coworkers' satisfaction and support, which corresponded to the “job resources” dimensions.

Factor 3 covered organizational context variables Factor 4 focused on personal fulfillment and alignment with work, which is congruent with Self-Determination Theory (SDT) and Person-Environment Fit (P-E Fit) (Table 2).

**Table 2.** Exploratory Factor Analysis Results.

Factor	Items Loaded	Variance Explained (%)	Total Variance Explained (%)
Workplace Demands	Workload, Shift Length	32.4	32.4
Job Satisfaction & Supervisor Support	Satisfaction with role, peer/supervisor support	24.6	57
Burnout & Mental Health	Emotional exhaustion, depersonalization	15.2	72.2
Career Development	Promotion opportunities, recognition	10.0	82.2

These composite factors adequately describe the major features of nurses' work perspectives related to burnout. Cronbach's Alpha was used to test the questionnaire's reliability, and the value of 0.85 indicated a high level of internal consistency.

(Table 2) Exploratory Factor Analysis Results for Nurse Survey (*N* = 345).

To further analyze the data and assess the study hypotheses, we used a combination of exploratory factor analysis (EFA), cluster analysis, and logistic regression. All quantitative analyses were conducted with SPSS.

The EFA results, including KMO, Bartlett's test, and factor loadings, guided the clustering and regression stages by validating that survey items grouped into significant dimensions aligned with theoretical constructs (demands, resources, etc.), affirming the construct validity of our measures.

### 4.6 Cluster analysis

A K-means clustering technique was used to divide nurses into homogeneous groups based on burnout risk, using important survey variables such as workload perception, job satisfaction, and support levels. K-means was chosen over alternatives such as latent class or hierarchical clustering because of its efficiency and practicality in interpreting continuous data in a large sample. We tested solutions with 2 to 5 clusters using the elbow approach and silhouette coefficients. The three-cluster design was chosen for its optimal blend of simplicity and internal cohesiveness.

Table 3 presents the characteristics of each cluster: Cluster 1 (Overworked & Exhausted) representing about 40 % of the sample, included nurses experiencing high workload and stress, low job satisfaction, and poor supervisor support. Cluster 2 (Struggling but Supported) comprising around 35 % of the sample, reported moderate burnout with some perceived support and moderate satisfaction. Cluster 3 (Resilient & Satisfied) comprising 25 % of the sample, demonstrated low burnout risk, high satisfaction, strong support, and manageable workloads. These clusters effectively stratified burnout risk: for instance, nurses in Cluster 1 were significantly more likely to report considering leaving their job and feeling emotionally exhausted, whereas Cluster 3 nurses were more engaged and content. These cluster assignments were used as the outcome variable in the regression analysis (Cluster 3 = reference group) (Table 3).

**Table 3.** Summarizes the characteristics of each cluster.

Cluster	% of Nurses	Burnout Level	Job Satisfaction	Workload	Supervisor Support
Cluster 1 (Overworked & Exhausted)	40 %	High (4.2/5)	Low (2.5/5)	High	Low
Cluster 2 (Struggling but Supported)	35 %	Moderate (3.1/5)	Moderate (3.4/5)	Medium	Medium
Cluster 3 (Resilient & Satisfied)	25 %	Low (2.1/5)	High (4.3/5)	Low	High

#### 4.6.1 Logistic regression

To test our hypotheses and identify significant predictors of burnout risk, we conducted multinomial logistic regression with cluster membership as the dependent variable. Specifically, we compared the odds of being in the High burnout cluster (Cluster 1) or Moderate (Cluster 2) vs the Low burnout cluster (Cluster 3), using workplace and demographic factors (e.g., workload, satisfaction, support, education, location, gender) as predictors. This enabled us to quantify the unique contribution of each variable while controlling for others. Regression results include estimated coefficients (β), standard errors, *p*-values, and odds ratios (OR = e^β) for interpretation of effect sizes. The overall model fit was strong (McFadden's pseudo-R^2^ = 0.42), indicating that a substantial portion of variance in burnout classification was explained. Table 4 provides full regression output and key findings are summarized in the Results section. All hypothesis tests used a significance level of α = 0.05 (two-tailed).

**Table 4.** Multinomial logistic regression model fit and performance.

Metric	Value
Log-Likelihood	−785.32
McFadden's Pseudo R^2^	0.42
Akaike Info. Criterion (AIC)	1585.64
Bayesian Info. Criterion (BIC)	1620.42
Overall Classification Accuracy	72 %

### 4.7 Linking theoretical framework to empirical analysis

The theoretical models underpinning this study- the Job Demands-Resources (JD-R) model, Conservation of Resources (COR) theory, Social Exchange Theory (SET), Person-Environment Fit (P-E Fit) theory, and Self-Determination Theory (SDT) guided the conceptualization, measurement, and analysis of burnout risk factors.

Exploratory Factor Analysis (EFA) identified key latent constructs reflecting theoretical domains: workload and shift length as job demands (JD-R), supervisor and peer support as job resources (JD-R, COR, SET), and personal fulfillment aligning with intrinsic motivation and value congruence (SDT, P-E Fit).

Cluster analysis was employed to classify nurses into burnout risk groups based on these factors, operationalizing the interplay between demands and resources as posited by JD-R and COR theories.

Multinomial logistic regression tested specific hypotheses derived from these theories, such as the protective role of supervisory support (COR, SET), the impact of workload on burnout (JD-R), and the influence of personal and organizational fit and autonomy (P-E Fit, SDT) on burnout risk.

This integrated approach ensured that the theoretical constructs were empirically examined, allowing for a robust test of the study hypotheses grounded in established burnout frameworks.

## 5 Results

### 5.1 Clusters of burnout risk and principal predictors

The clustering analysis identified three distinct burnout risk profiles among nurses. To determine which factors significantly predicted cluster membership, we applied a multinomial logistic regression model, with Low burnout (Cluster 3) as the reference category. The model assessed the likelihood of nurses belonging to the High (Cluster 1) or Moderate (Cluster 2) burnout groups based on various predictors. Table 4 presents the overall model fit and classification metrics, while Table 5 details the regression coefficients for each predictor. For clarity, our interpretation focuses on comparisons between High vs. Low burnout risk (Cluster 1 vs. 3), directly addressing hypotheses H1–H6, with additional notes on the Moderate group where applicable.

**Table 5.** Logistic regression coefficients predict burnout risk cluster membership (Cluster 3: Low burnout is reference).

Predictor	β (High vs. Low) [95 % CI]	β (Moderate vs. Low) [95 % CI]	p-value
Intercept	1.92 (1.45, 2.39)	0.87 (0.55, 1.19)	0.001
Workload	0.78 (0.65, 0.91)	0.42 (0.29, 0.56)	< 0.001
Shift Length	0.52 (0.36, 0.68)	0.31 (0.18, 0.44)	0.002
Job Satisfaction	–0.61 (−0.72, −0.49)	−0.45 (−0.57, −0.32)	< 0.001
Supervisor Support	−0.50 (−0.63, −0.38)	−0.32 (−0.47, −0.18)	0.004
Education – Bachelor's (vs. Diploma)	−0.35 (−0.50, −0.20)	−0.22 (−0.38, −0.06)	0.020
Education – Master's/PhD (vs. Diploma)	−0.58 (−0.75, −0.41)	−0.40 (−0.57, −0.23)	0.008
Location – Rural (vs. Urban)	0.45 (0.15, 0.75)	0.21 (0.05, 0.37)	0.015
Gender – Female (vs. Male)	0.22 (0.08, 0.36)	0.14 (0.02, 0.26)	0.032

(Notes: β = logistic regression coefficient; OR = odds ratio = e^β; p*<0.05, **p**<0.01, n.s. = not significant.)

The Pseudo R^2^ value of 0.42 suggests that the model explains approximately 42 % of the variance in burnout group membership, indicating a good fit. The AIC (1585.64) and BIC (1620.42) values further support the adequacy of the model, as lower values are indicative of better model performance. Moreover, the model achieved an overall classification accuracy of 72 %, which is considerably higher than the expected 33 % by chance for three equally sized groups, highlighting the model's effectiveness in distinguishing burnout risk categories (Table 4).

A positive β indicates higher odds of being in the respective burnout group (High or Moderate) compared to Low, for each unit increase in the predictor or relative to the reference group for categorical variables.

Several predictors show statistically significant effects on the burnout risk category:

#### 5.1.1 H1 (Workload)

Higher perceived workload is the strongest predictor of being in a higher burnout category. For each one-unit increase in workload score, the odds of a nurse being in the High burnout group (vs. Low burnout) increase by a factor of e^0.78 ≈ 2.18 (a 118 % increase), holding other factors constant. Similarly, workload significantly differentiates Moderate vs. Low burnout (β = 0.42, *p* < 0.001), supporting H1 and aligning with the JD-R model's prediction that excessive job demands drive burnout. Extended work hours also contribute to burnout, though to a lesser degree. Each additional 10 h of work per week (roughly one extra shift) is associated with an 8 % increase in odds of high burnout (*p* = 0.023). Moreover, longer shift durations raise the odds of High vs. Low burnout by a factor of e^0.52 ≈ 1.68 (a 68 % increase). These findings indicate that very long shifts exacerbate exhaustion and burnout.

#### 5.1.2 H2 (Job Satisfaction)

Job satisfaction is a protective factor against burnout. A one-unit increase in overall job satisfaction score corresponds to a β of −0.61 for High vs. Low burnout (OR = e^−0.61 ≈ 0.54), indicating about 46 % lower odds of being in the High burnout group. Similarly, higher satisfaction reduces the odds of Moderate vs. Low burnout (β = −0.45). Nurses satisfied with their roles, work conditions, and recognition were significantly less likely to experience high burnout. This confirms H2 and underscores that fostering job satisfaction through improved work environment and recognition can substantially reduce burnout risk, consistent with the JD-R model (job resources mitigate burnout) and P-E Fit/SDT theories (alignment and need fulfillment promote well-being).

#### 5.1.3 H3 (Supervisor Support)

Strong supervisor support significantly predicts lower burnout risk. Nurses who feel supported by their supervisors have a reduced likelihood of being in a higher burnout category (β = −0.50 for High vs. Low, OR ≈ 0.61; *p* = 0.004), corresponding to roughly a 39 % reduction in odds of High burnout per step increase in support rating. This finding supports H3 and aligns with COR theory (supervisory support conserves nurses' emotional energy) and SET (supportive treatment fosters resilience and engagement). Notably, supervisor support differentiates more strongly between High vs. Low burnout groups than Moderate vs. Low (the latter was not statistically significant, *p* = 0.18), suggesting lack of support particularly distinguishes the most burned-out nurses from those less affected.

#### 5.1.4 H4 (Education Level)

Educational level showed an inverse relationship with burnout risk, confirming H4. Nurses with a bachelor's degree had significantly lower odds of high burnout compared to those with only a nursing diploma (β = −0.35, *p* = 0.020), while those with a master's degree or PhD exhibited an even stronger protective effect (β = −0.58, *p* = 0.008). For example, having a master's/PhD versus a diploma corresponds to an OR of e^–0.58 ≈ 0.56 for High vs. Low burnout, representing about a 44 % reduction in odds. Highly educated nurses may benefit from better coping strategies, roles better aligned with their skills (P-E Fit), or greater job control (SDT's autonomy need), which collectively protect against burnout.

#### 5.1.5 H5 (Location Urban vs. Rural)

Work location emerged as a significant factor (H5). Nurses in rural settings had higher odds of being in the High burnout group compared to those in urban areas (β = 0.45, *p* = 0.015), corresponding to about e^0.45 ≈ 1.57 times the odds (a ∼57 % increase). This likely reflects resource constraints or other stressors common in rural healthcare environments. The effect was smaller but marginally significant for Moderate vs. Low burnout (β = 0.21), indicating the rural impact is strongest at the highest burnout levels.

#### 5.1.6 H6 (Gender)

Female nurses were more likely than males to experience higher burnout levels, supporting H6. For High vs. Low burnout, female gender had β = 0.22 (*p* = 0.032), corresponding to about e^0.22 ≈ 1.25 times higher odds (25 % increase), controlling for other factors. A similar, slightly smaller effect was seen for Moderate vs. Low burnout (β = 0.14). This suggests female nurses report higher burnout incidence, potentially due to gender-related work stressors or societal role pressures (**Table_5**). While our theoretical models did not directly address gender, this finding is consistent with prior observations in nursing literature.

The regression analysis confirms all hypothesized associations (H1–H6) in direction, with the majority attaining statistical significance. Elevated workloads and extended hours heighten the risk of burnout, but robust employment resources (such as satisfaction and support) and personal resources (like education) mitigate it. Rural practice and female gender were identified as risk factors for increased burnout in this context.

The multinomial logistic regression model accurately classified 72 % of nurses into burnout groups, with a good fit (Pseudo R^2^ = 0.42). Notably, the model had a high sensitivity for the High burnout group, with a recall of around 71 %, demonstrating its usefulness in identifying nurses at high risk of burnout (**Table_5**).

The confusion matrix (Table 6) demonstrates the model's robust classification efficacy, accurately recognizing 71 % of nurses in the High burnout category and 85 % in the Low burnout category.

**Table 6.** Model classification results (confusion matrix, showing percentage of nurses in each actual category classified into each predicted category).

Actual / Predicted	High Burnout (Cluster 1)	Moderate Burnout (Cluster 2)	Low Burnout (Cluster 3)
Cluster 1 – High	71 %	22 %	7 %
Cluster 2– Moderate	18 %	74 %	8 %
Cluster 3 – Low	5 %	10 %	85 %

In the High burnout category, the model attained a precision of 70 %, a recall of 71 %, and an F1-score of 69 %, demonstrating its efficacy in identifying nurses at heightened risk and informing targeted interventions.

## 6 Discussion

This study's findings align strongly with the Job Demands-Resources (JD-R) model, confirming that high workload and extended shifts are key contributors to nurse burnout in Albanian hospitals. Nurses managing high patient loads without sufficient support were more likely to fall into the “Overworked & Exhausted” cluster, an outcome consistent with prior research linking job strain with emotional exhaustion [34]. These results underscore the need for fair shift allocation, increased staffing levels, and improved nurse-to-patient ratios.

Supervisor support emerged as a crucial protective factor. Nurses who felt respected and supported by their supervisors reported lower emotional fatigue. This supports both the Social Exchange Theory (SET), which emphasizes perceived fairness and managerial responsiveness [37], and the Conservation of Resources (COR) theory, which identifies supervisory support as a psychological resource that helps conserve emotional energy and mitigate stress [36].

Job satisfaction was inversely associated with burnout, consistent with the Person-Environment Fit (P-E Fit) theory. When professional values align with organizational culture, nurses experience higher fulfillment and lower burnout risk [39]. Factors such as fair compensation, recognition, and opportunities for career advancement emerged as key to fostering satisfaction and reducing disengagement.

While burnout was not measured using a standardized scale, the findings closely align with theoretical constructs defined in major burnout frameworks, particularly the Self-Determination Theory (SDT). Conditions promoting competence (via access to professional development and higher education), relatedness (through supportive supervision and team cohesion), and autonomy (nurses' involvement in decision-making) were associated with lower burnout levels, indirectly validating SDT principles. Empirically, nurses with advanced degrees (e.g., master's) exhibited greater resilience, consistent with prior research linking higher education to enhanced coping skills, autonomy, and professional competence, factors that reduce burnout risk [8,13,15].

Our analysis also revealed rural–urban disparities, with nurses in rural areas experiencing higher burnout levels, even when controlling for workload and supervisor support. This supports previous findings on the challenges of rural healthcare including resource shortages, professional isolation, and staffing instability [27,28].

Gender differences were observed, with female nurses reporting slightly higher burnout than males. Although modest, this difference aligns with existing evidence that women may face additional stressors related to societal roles and family responsibilities. To address these challenges, gender-sensitive workplace policies like flexible scheduling, parental leave, and on-site childcare are recommended. However, it is important to note that burnout affects all genders, and interventions should be inclusive.

Beyond individual implications, these findings hold relevance for health systems in low-resource settings like Albania. Investing in supportive structures, fair employment practices, and ongoing professional development is essential to strengthening workforce resilience and improving healthcare delivery through better staff well-being [45].

### 6.1 Future research

This study offers several opportunities for future research on nurse burnout:

• Longitudinal studies are needed to monitor burnout trajectories and assess the impact of health policy changes or wellness initiatives on nurse well-being.
• Employing mixed-method designs that combine survey data with qualitative insights from interviews or focus groups would provide a more comprehensive understanding of burnout's causes and consequences.
• Designing intervention studies to evaluate the impact of strategies such as supervisor training, peer support programs, or shift scheduling reforms.
• Examining whether organizational changes like improved nurse-to-patient ratios or mentorship programs result in significant burnout reduction.
• Crucially, future research should incorporate validated burnout scales, such as the Maslach Burnout Inventory (MBI) or the Copenhagen Burnout Inventory (CBI), alongside the indirect measures used in this study. This combined approach would enhance construct validity, facilitate international contextual comparisons, and contribute to the development of a culturally adapted burnout assessment tool tailored for Albania.
• Finally, future studies should explore associations between nurse burnout and patient care indicators, including satisfaction and safety outcomes, as well as investigate the role of nursing education reforms particularly in stress resilience training and occupational preparedness as preventive measures against burnout [46].

## 7 Study limitations

While this study offers important insights into nurse burnout, several limitations must be acknowledged.

First, as a cross-sectional study, it cannot establish causal relationships, since it captures data at a single point in time. It remains unclear whether burnout causes nurses to perceive higher workloads or if demanding workloads directly increase burnout risk. Future research should utilize longitudinal designs to monitor changes over time and assess the effectiveness of targeted interventions.

Second, reliance on self-reported data introduces potential response bias, including social desirability and recall inaccuracies. Although self-reports provide valuable subjective perspectives, incorporating objective indicators such as administrative logs, absenteeism rates, or patient outcomes would strengthen future analyses and triangulate findings.

Third, burnout was assessed indirectly using empirically grounded antecedents rather than a standardized instrument such as the Maslach Burnout Inventory (MBI) or Copenhagen Burnout Inventory (CBI). While this approach allowed for a concise and context-sensitive survey design, it inherently limits the ability to capture all core dimensions of burnout particularly depersonalization and diminished personal accomplishment. As such, the findings should be interpreted as indicative of burnout risk rather than a direct diagnostic assessment. Future studies should incorporate validated burnout instruments to improve construct validity and enable comparability across settings.

Fourth, the study was conducted in a single regional tertiary hospital. However, due to national-level standardization of staffing, budgeting, and service delivery in Albanian public healthcare, these findings may be analytically generalized to similar public hospitals, especially those outside the capital. Caution remains necessary when applying these results to private institutions or specialized tertiary centers, which may operate under different models.

Finally, the questionnaire was translated and culturally adapted into Albanian. Despite efforts to preserve the original meaning, subtle differences in interpretation may have affected participants' understanding of certain items, potentially influencing the results.

## 8 Conclusion

This study identifies key contributors to nurse burnout in a regional public hospital in Albania, including excessive workloads, insufficient managerial support, and misalignment between nurses' responsibilities and their professional values. While acknowledging limitations such as reliance on self-reported data and the absence of a validated burnout instrument, the findings offer meaningful insights for healthcare practice and policy development.

Burnout is not merely an individual issue, but a systemic challenge shaped by organizational structures and workforce management. Addressing it requires targeted strategies such as revising staffing models, implementing support and mentorship programs, aligning tasks with nurses' competencies, and promoting continuous professional development.

Reducing burnout is essential to ensure nurse retention, enhance patient safety, and improve care quality. Sustained investment in supportive workplace cultures, validated assessment tools, and ongoing research will be critical for strengthening the resilience of the nursing workforce in Albania and similar healthcare systems.

## Standards of reporting

The study followed the Strengthening the Reporting of Observational Studies in Epidemiology (STROBE) guidelines.

## CRediT authorship contribution statement

**Edona Haxhija:** Writing – review & editing, Writing – original draft, Software, Investigation, Data curation. **Drita Kruja:** Writing – original draft, Validation, Project administration, Methodology, Formal analysis, Data curation. **Zamira Shabani:** Writing – review & editing, Conceptualization.

## Consent for publication

Informed consent was obtained from all participants involved in the study.

## Ethical approval and consent to participate

This study was conducted in accordance with the Declaration of Helsinki and approved by the Ethics Committee of the Faculty of Natural Sciences, Department of Clinical Subjects, University of Shkodra (Protocol No. 89/1, dated 29 May 2024, Shkodër, Albania).

## Funding

This research did not receive any specific grant from funding agencies in the public, commercial, or not-for-profit sectors.

## Declaration of competing interest

The authors declare no competing interests, financial or otherwise.

## Data Availability

The data supporting the findings of this article are available from the corresponding author upon reasonable request.

## Appendix A Questionnaire

### A.1 Appendix A: Nurse Satisfaction Survey

**Demographic Information.**

**Gender:** Female / Male; **Age:** ______; **Department/Unit:** ________; **Years of experience as a nurse:** ______; **Place of residence:** Urban / Rural; **Education level:** Bachelor's degree/ Professional Master's degree/ Scientific Master's degree/ Doctoral degree.

**Survey Questions**

1. **Are you satisfied with your role as a nurse in this workplace?**

a) Very satisfied b) Satisfied c) Neutral d) Dissatisfied e) Very dissatisfied

2. **Are you satisfied with your junior colleagues?**
  a) Very satisfied b) Satisfied c) Neutral d) Dissatisfied e) Very dissatisfied
3. **Are you satisfied with your senior colleagues?**
  a) Very satisfied b) Satisfied c) Neutral d) Dissatisfied e) Very dissatisfied
4. **Are you satisfied with colleagues who have similar years of experience as you?**
  a) Very satisfied b) Satisfied c) Neutral d) Dissatisfied e) Very dissatisfied
5. **Are you satisfied with the equipment available in your workplace?**
  a) Very satisfied b) Satisfied c) Neutral d) Dissatisfied e) Very dissatisfied
6. **Are you satisfied with your head nurse?**
  a) Very satisfied b) Satisfied c) Neutral d) Dissatisfied e) Very dissatisfied
7. **Are you satisfied with your salary?**
  a) Very satisfied b) Satisfied c) Neutral d) Dissatisfied e) Very dissatisfied
8. **Overall, are you satisfied with your workplace?**

a) Very satisfied b) Satisfied c) Neutral d) Dissatisfied e) Very dissatisfied

9. **Do you believe there is job security in your workplace?**
  a) Yes b) No
10. **If there is a lack of job security, do you feel pressured because of it?**
  a) Yes b) No
11. **Are the mission and vision of your workplace clear to you?**
  a) Yes b) No
12. **Is your workplace aligned with your personal mission and values?**
  a) Yes b) No
13. **Are there opportunities for promotion in your workplace?**
  a) Yes b) No
14. **Is there sufficient staffing in your workplace?**

a) Yes b) No

15. **If staffing is insufficient, do you feel pressured?**

a) Yes b) No

16. **Are there times when you find it difficult to deal with patients?**
  a) Yes b) No
17. **Are there times when you find it difficult to deal with patients' family members?**
  a) Yes b) No
18. **Do you receive sufficient support from your colleagues?**

a) Yes b) No

19. **Do you receive sufficient support from your head nurse?**
  a) Yes b) No
20. **Are you respected by your colleagues?**

a) Yes b) No

21. **Do your colleagues listen to your work-related comments or concerns?**

a) Yes b) No

22. **Does your head nurse listen to your work-related concerns?**
  a) Yes b) No
23. **Are you proud to be a nurse?**

a) Yes b) No

24. **Are you proud of your work?**
  a) Yes b) No.
